# Indirect comparison of deucravacitinib and other systemic treatments for moderate to severe plaque psoriasis in Asian populations: A systematic literature review and network meta‐analysis

**DOI:** 10.1111/1346-8138.17448

**Published:** 2024-11-11

**Authors:** Tsen‐Fang Tsai, Yayoi Tada, Camy Kung, Yichen Zhong, Allie Cichewicz, Katarzyna Borkowska, Tracy Westley, Renata M. Kisa, Yu‐Huei Huang, Xing‐Hua Gao, Seong‐Jin Jo, April W. Armstrong

**Affiliations:** ^1^ Department of Dermatology National Taiwan University Hospital Taipei Taiwan; ^2^ Department of Dermatology Teikyo University School of Medicine Tokyo Japan; ^3^ Global Medical Affairs Bristol Myers Squibb Taipei Taiwan; ^4^ Worldwide HEOR Bristol Myers Squibb Princeton New Jersey USA; ^5^ Evidera Evidence Synthesis, Modeling and Communication Waltham Massachusetts USA; ^6^ Evidence Synthesis, Modeling and Communication PPD Poland Warsaw Poland; ^7^ Bristol Myers Squibb Princeton New Jersey USA; ^8^ Department of Dermatology Chang Ghang Memorial Hospital and Chang Gung University Taoyuan Taiwan; ^9^ Department of Dermatology First Hospital of China Medical University Shenyang China; ^10^ Department of Dermatology Seoul National University College of Medicine Seoul South Korea; ^11^ Department of Medicine University of California Los Angeles Los Angeles California USA

**Keywords:** Apremilast, biologics, deucravacitinib, network meta‐analysis, psoriasis

## Abstract

Expanding the systemic treatment options for patients with psoriasis, deucravacitinib, an oral, selective, allosteric tyrosine kinase 2 inhibitor is approved in the United States, European Union, China, Japan, Taiwan, Korea, and other countries for the treatment of adults with moderate to severe plaque psoriasis who are candidates for systemic therapy. Evidence suggests the comparative efficacy of systemic therapies may be different in Asian versus White patients. This systematic review and network meta‐analysis (NMA) evaluated the clinical efficacy associated with deucravacitinib and other biologic or non‐biologic systemic treatments for moderate to severe plaque psoriasis in Asian populations. Electronic databases were searched to identify randomized trials of the interventions of interest. Multinomial random effects models adjusting for baseline placebo risk were used to estimate Psoriasis Area and Severity Index (PASI) responses at weeks 10–16. Of 8596 studies identified, 20 were included in the NMA. The estimated PASI 75 and 90 (95% credible interval) response rates for deucravacitinib were estimated to be 66% (49%–80%) and 40% (24%–58%) in Asian populations, notably higher than placebo (6% [4%–9%] and 1% [0.8–2%]) and apremilast (24% [12%–40%] and 9% [4%–20%]). No statistically significant difference was observed in PASI 75 and 90 responses between deucravacitinib and adalimumab, certolizumab pegol, infliximab, ustekinumab, and tildrakizumab. Deucravacitinib demonstrated robust efficacy in the Asian population, with PASI 75 and 90 responses comparable to some biologics. Deucravacitinib provides a convenient oral therapy with efficacy similar to several biologic therapies.

## 1 INTRODUCTION

Systemic treatment options for patients with moderate to severe psoriasis include conventional therapies, targeted small molecules and biologics. The biologics include the first‐generation antitumor necrosis factor (anti‐TNF) inhibitors and interleukin (IL) 12/23 inhibitors as well as the second‐generation IL‐17 and IL‐23 inhibitors. Targeted small molecules include apremilast and the recent addition, deucravacitinib, an oral, selective, allosteric tyrosine kinase 2 (TYK2) inhibitor. Deucravacitinib is approved in the United States, European Union, Japan, China, Taiwan, Korea, and other countries for the treatment of adults with moderate to severe plaque psoriasis who are candidates for systemic therapy.^1^, ^2^, ^3^ In two global, phase 3 clinical trials (POETYK PSO‐1 and PSO‐2), deucravacitinib demonstrated superior efficacy compared with apremilast.^4^, ^5^

The number of available treatments makes direct comparisons of all possible combinations unfeasible; therefore, systematic literature reviews (SLRs) and network meta‐analyses (NMAs) are used to identify published literature and to evaluate the feasibility of indirectly comparing the relative effects of various therapies through a common comparator (i.e, placebo). Evidence suggests that the comparative efficacy of systemic therapies may be different in Asian patients versus those of other ethnicities due to demographic and patient characteristics, psoriatic disease characteristics, clinical practice, and lifestyle differences such as smoking and alcohol use.^6^, ^7^ However, NMAs specifically focused on these differences in the Asian population have not been published.

The objective of this SLR and NMA was to estimate the comparative effectiveness of deucravacitinib relative to other systemic treatments in the Asian patient population with moderate to severe plaque psoriasis (particularly patients from the East Asia region).

## 2 METHODS

The SLR was performed following a prespecified protocol (available through the corresponding author) and conducted in accordance with the Cochrane Handbook for Systematic Reviews of Interventions, and following the standards required by the National Institute for Health and Care Excellence (NICE).

### 2.1 Study selection and data extraction

Electronic databases were searched to identify English‐language, randomized controlled trials (RCTs) published from database inception through January 3, 2023 (Tables S1‐S6). Conference proceedings from 2019 to 2022 and clinical trial registries were also searched. The methods used to evaluate studies was reported previously.^4^ Eligible studies were RCTs of systemic treatments conducted in Asia (including East Asia – China, Japan, Korea, and Taiwan) or with patients considered to be of Asian descent (i.e., Asian ethnicity). Eligible studies in the SLR included adults with moderate to severe psoriasis who reported Psoriasis Area and Severity Index (PASI) responses of 50%, 75%, 90%, or 100% reduction from baseline (PASI 50, PASI 75, PASI 90, or PASI 100, respectively). Complete inclusion and exclusion criteria are listed in Table 1. Interventions and comparators were limited to only those treatments approved as of January 2023 in China, Japan, Korea, and Taiwan.

**TABLE 1:** Study selection criteria.

Category	Inclusion criteria	Exclusion criteria
Population	Asian adult patients (aged ≥18 years) with moderate to severe^a^ plaque PsO who are candidates for systemic therapies	Studies on patients with forms of plaque PsO other than moderate to severe Studies on pediatric patients Studies that only focus on the treatment of PsA. This does not include studies that are in PsO populations with comorbid PsA unless 100% of patients also have PsA. Studies on patients with palmoplantar pustulosis
Interventions	Biologics: Tumor necrosis factor inhibitors Certolizumab pegol 400 mg or 200 mg Q2W (with loading dose of 400 mg) Adalimumab 40 mg EOW (with loading dose of 80 mg) Etanercept 50 mg QW, 50 mg BIW or 25 mg BIW Infliximab 5 mg/kg Q8W Interleukin‐17 inhibitors Bimekizumab 320 mg Q4W Brodalumab 210 mg Q2W Ixekizumab 80 mg Q2W (with loading dose of 160 mg) Secukinumab 150 mg or 300 mg Q4W Interleukin‐23 inhibitors Guselkumab 100 mg Q8W Risankizumab 75 or 150 mg Q12W Tildrakizumab 100 mg or 200 mg Q12W Interleukin‐12/Interleukin‐23 inhibitors Ustekinumab 45 mg or 90 mg Q12W Targeted small molecules: Tyrosine kinase 2 inhibitors Deucravacitinib 6 mg OD Apremilast 30 mg BID Conventional small molecules^b^: Methotrexate 7.5 mg–25 mg Cyclosporine 2.5–5 mg/kg/day Acitretin 0.4 mg/kg Etretinate	Studies that do not include a treatment arm with any of the selected comparators of interest
Comparisons	Placebo Best supportive care Any of the above therapies	N/A
Outcomes	Efficacy: Proportion of patients with PASI 50 response (at 10–16, 24–28, 44–60 weeks) Proportion of patients with PASI 75 response (at 10–16, 24–28, 44–60 weeks) Proportion of patients with PASI 90 response (at 10–16, 24–28, 44–60 weeks) Proportion of patients with PASI 100 response (at 10–16, 24–28, 44–60 weeks)	Organ‐specific PASI (i.e., nail PASI) Other outcomes or time points not listed as of interest
Study designs	RCTs (phase 2, 3, 4) (including follow‐up studies of RCTs)	Observational/real‐world evidence studies Single‐arm trials Phase 1 trials SLRs/NMAs^c^ Pooled analyses of trials^c^
Subgroups	Biologic naive Biologic exposed Asian patients based on geography and/or race	N/A
Other limits	Only English‐language articles/conference abstracts	Journal articles and conference abstracts not available in English
	No limitation for peer‐reviewed publications, last 3 years for conference abstracts	Studies published outside the time frame of interest
	Only trials conducted in Asian countries will be included. Subgroups of Asian patients from global trials will also be considered for inclusion	Trials conducted outside of Asia without subgroup data for Asian patients

Abbreviations: BID, twice a day; BIW, twice weekly; DLQI, Dermatology Life Quality Index; EOW, every other week; IGA, Investigator's Global Assessment; N/A, not applicable; NMA, network meta‐analysis; OD, once daily; PASI, Psoriasis Area and Severity Index; PGA, Physician Global Assessment; PsA, psoriatic arthritis; PsO, psoriasis; PSSD, Psoriasis Symptoms and Signs Diary; QW, every week; Q2W, every 2 weeks; Q4W, every 4 weeks; Q8W, every 8 weeks; Q12W, every 12 weeks; RCT, randomized controlled trial; SLR, systematic literature review; TNF, tumor necrosis factor; TYK2, tyrosine kinase 2.

^a^ If “moderate to severe” was mentioned, then that was sufficient criteria for inclusion regardless of the definition. However, if “moderate to severe” was not mentioned, a decision was made with clear documentation based on the PGA, PASI, body surface area (BSA), and Dermatology Life Quality Index (DLQI) criteria (PGA ≥3, PASI ≥10, BSA ≥10, or DLQI ≥10).

^b^ Any dose of systemic conventional small molecule treatments was included, as doses are often modified or titrated.

^c^ The list of included studies from each publication was reviewed to identify any additionally relevant RCTs not otherwise captured by the database searches. These publications themselves were not included in the systematic literature review unless unique data were available that was not published elsewhere from the individual trials.

Study selection followed a 2‐stage screening process. First, titles and abstracts identified by the search strategies were independently reviewed by two researchers to determine eligibility according to the inclusion and exclusion criteria. A third reviewer resolved discrepancies, if necessary. Second, all full‐text articles deemed eligible in the first stage were reviewed independently by two researchers. The studies at the second stage met all the protocol‐specified inclusion criteria and had no exclusion criteria. Discrepancies were resolved by a third reviewer, as needed.

Data were extracted by one researcher and independently validated by a second researcher. Data elements of interest were study characteristics (e.g., author, publication year, sponsor, study objectives, sample size), patient characteristics (e.g., age, gender, comorbidities), treatment regimen, and outcomes related to efficacy and safety. Studies published as multiple articles (e.g., interim and/or final results) were extracted as one study. Data for subgroup analyses (e.g., previous use of biologics) were extracted by the treatment arm in each trial, if available.

### 2.2 Feasibility assessment

To ensure the two main NMA assumptions of consistency and similarity were met across the included trials, a feasibility assessment was performed. The characteristics of the trials identified in the SLR and connected in the network (i.e., study design, patient characteristics, interventions and comparators, and outcomes) were evaluated to determine if they were sufficiently similar to be synthesized quantitatively, and whether any imbalances in potential effect modifiers existed. Evidence of effect modification was identified through the evaluation of subgroup analyses of the included trials, published literature, and previous NICE technology appraisals for moderate to severe plaque psoriasis. Effect modifiers were validated by clinical experts.

### 2.3 NMA methodology

This study followed PRISMA Reporting Guidelines for meta‐analysis (Table S7). This methodology was described previously. In brief, this NMA used a multinomial (probit) REZ model with random effects, adjusted for baseline placebo risk (i.e., placebo response) to estimate the probability for achieving a PASI response for each treatment over 10–16 weeks, the number needed to treat (NNT) to achieve one additional PASI 75 response, and the relative odds of achieving a PASI response between treatments.

For each treatment, the NNT to achieve one additional PASI 75 or PASI 90 response relative to placebo was calculated as the reciprocal of the difference in estimated PASI 75 response rates between the active treatment and placebo.

## 3 RESULTS

**FIGURE 1:**
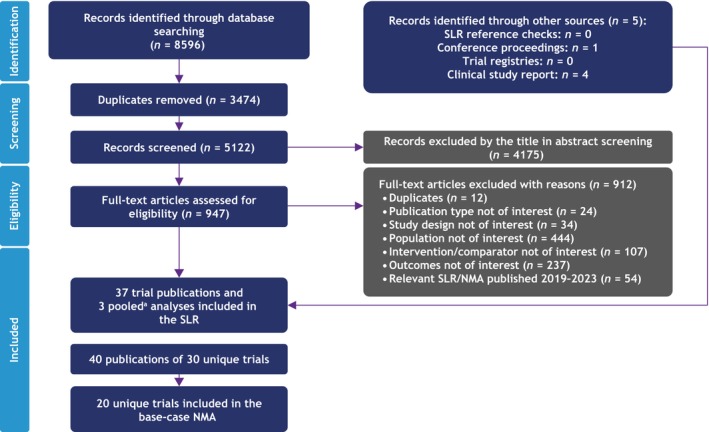
Systematic literature review (SLR) attrition diagram. ^a^Pooled analyses of randomized controlled trial were not included in the SLR unless unique data were available that were not published elsewhere. NMA, network meta‐analysis.

Of 8596 records identified by the searches, 20 unique RCTs were included in the NMA (Figure 1); 15 were exclusively Asian studies, while five were Asian subgroups of larger, global studies. Most studies enrolled or reported on Japanese patients (10 studies), followed by Chinese (seven studies), South Korean (five studies), and Taiwanese patients (four studies). While several trials included patients from more than one country, only one trial (ERASURE) reported data for two distinct subgroups of Asian patients (Taiwanese and Korean). Study populations were generally similar with respect to age and sex. Patients were, on average, between 32.0 and 53.3 years old, and most participants in each study were male. The mean body weight ranged from 65.8 to 80.5 kg; however, most studies reported a mean weight of less than 75 kg (Table 2).

**TABLE 2:** Study and patient characteristics included in the NMA.

Trial (phase)	Total patients No.	Primary endpoint weeks	Severity definition	Intervention(s) and comparators	Age, mean (SD) years	Body weight mean (SD) kg	Disease duration years	Prior biologic therapy, %	Race/ethnicity, %
Asahina^8^ 2010 (2/3)	169	16	PASI ≥12, BSA ≥10%	Adalimumab 80 mg at weeks 0, then 40 mg Q2W	44.2 (14.3)	67.4 (9.9)	14.0	0^a^	Japanese: 100
				Placebo	43.9 (10.8)	71.3 (15.3)	15.5	0^a^	Japanese: 100
Cai^9^ 2017 (3)	425	12	NR	Adalimumab 80 mg at weeks 0, then 40 mg Q2W	43.1 (11.9)	69.7 (12.4)	14.8	0^a^	Chinese: 100
				Placebo	43.8 (12.5)	67.0 (10.6)	15.8	0^a^	Chinese: 100
CAIN457A2318^10^ (3b)	441	12	PASI ≥12, BSA ≥10%, IGA ≥3	Secukinumab 300 mg QW to week 4, then Q4W	39.0 (11.6)	73.3 (14.2)	15.0	14.9	Chinese: 100
				Secukinumab 150 mg QW to week 4, then Q4W	40.5 (10.8)	72.7 (15.5)	16.2	21.8	Chinese: 100
				Placebo	38.7 (10.3)	72.6 (13.3)	14.8	20.9	Chinese: 100
CLEAR^11^, ^12^ (3)	676	16	PASI ≥12, BSA ≥10%, mIGA ≥3	Secukinumab 300 mg QW to week 4, then Q4W	39.1 (15.1)	NR	12.39	17.4	Korean/Taiwanese: 80
				Ustekinumab 45 or 90 ng at weeks 0 and 4, then Q12W	39.8 (14.1)	NR	12.59	17.9	Korean/ Taiwanese: 80
ERASURE Japanese subgroup (3)	87	12	PASI ≥12, BSA ≥10, mIGA ≥3	Secukinumab 300 mg QW to week 4, then Q4W	51.9 (11.8)	76.5 (14.4)	15.6	20.7	Japanese: 100
				Secukinumab 150 mg QW to weeks 4, then Q4W	48.2 (13.1)	74.2 (16.5)	15.6	17.2	Japanese: 100
				Placebo	50.2 (13.6)	72.6 (18.9)	14.1	20.7	Japanese: 100
ERASURE^12^ Taiwanese subgroup (3)	738	12	PASI ≥12, BSA ≥10%, mIGA ≥3	Secukinumab 300 mg QW to week 4, then Q4W	38.1 (12)	74.7 (13.4)	13.6	Anti‐TNF: 25 Anti‐IL 12/23: 12.5 Other: 12.5	Taiwanese: 100
				Secukinumab 150 mg QW to week 4, then Q4W	39.5 (10.9)	71.5 (15.7)	14.5	Anti‐TNF: 25 Anti‐IL 12/23: 25 Other: 5	Taiwanese: 100
				Placebo	40.6 (10.8)	78.2 (20.0)	8.3	Anti‐TNF: 6.7 Anti‐IL 12/23: 13.3 Other: 6.7	Taiwanese: 100
I1F‐MC‐RHBH (3)		12	NR	Ixekizumab 160 mg at weeks 0 then 80 mg Q2W	NR	NR	NR	NR	Chinese: 100
				Placebo	NR	NR	NR	NR	Chinese: 100
LOTUS (3)	322	12	PASI ≥12, BSA ≥10%	Ustekinumab 45 mg at weeks 0, 4, and 16	40.1 (12.4)	69.6 (11.6)	14.6	11.9	Chinese: 100
				Placebo	39.2 (12.2)	69.6 (12.1)	14.2	6.8	Chinese: 100
Igarashi^13^ 2012 (2/3)	158	12	PASI ≥12, BSA ≥10%	Ustekinumab 45 mg at weeks 0 and 4, then Q12W	45.0	73.2 (15.4)	15.8	1.6	Japanese: 100
				Ustekinumab 90 mg at weeks 0 and 5, then Q12W	44.0	71.1 (14.0)	17.3	0	Japanese: 100
				Placebo	49.0	71.2 (10.9)	16.0	0	Japanese: 100
Nakagawa^14^ 2016 (2)	151	12	PASI ≥12, BSA ≥10%	Brodalumab 210 mg at weeks 0, 1, and 2, then Q2W	46.4 (11.8)	72.6 (15.9)	15.0	13.5	Japanese: 100
				Placebo	46.6 (10.8)	72.2 (15.2)	16.9	7.9	Japanese: 100
Ohtsuki, 2017 (2b)	254	16	PASI ≥12, BSA ≥10%	Apremilast 30 mg BID	51.7 (12.7)	70.1 (13.0)	13.9	2.4	Japanese: 100
				Placebo	48.3 (12.0)	68.5 (13.8)	12.4	4.8	Japanese: 100
Ohtsuki^15^ 2018 (3)	192	16	PASI ≥12, BSA ≥10%, IGA ≥3	Guselkumab 100 mg at weeks 0 and 4, then Q8W	47.8 (11.1)	74.3 (16.0)	14.4	17.5	Japanese: 100
				Placebo	48.3 (10.6)	71.6 (14.0)	13.7	15.6	Japanese: 100
POETYK PSO‐1^16^ (3)	666	16	PASI ≥12, BSA ≥10%, sPGA ≥3	Deucravacitinib 6 mg OD	32.0 (13.1)	NR	12.12	22	Chinese, Japanese, Korean, Taiwanese: 100
				Apremilast 30 mg BID	36.2 (11.3)	NR	12.08	22.6	Asian: 100
				Placebo	36.3 (12.3)	NR	14.13	38.0	Asian: 100
POETYK PSO‐3 (3)	220	16	PASI ≥12, BSA ≥10%, sPGA ≥3	Deucravacitinib 6 mg OD	40.3 (12.9)	77.5 (15.8)	13.2	46.7	Chinese: 80.8 Korean: 19.2
				Placebo	41.2 (12.3)	74.5 (14.4)	13.9	47.7	Chinese: 83.8 Korean: 16.2
PEARL (3)	121	12	PASI ≥12, BSA ≥10%	Ustekinumab 45 mg at weeks 0, 4, 16	40.9 (12.7)	73.1 (12.7)	11.9	21.3	Taiwanese/Chinese: 49.2 Korean: 50.8
				Placebo	40.4 (10.1)	74.6 (13.0)	13.9	15.0	Taiwanese/Chinese: 50 Korean: 50
reSURFACE 1, Japanese subgroup analysis^17^ (3)	158	12	PASI ≥12, BSA ≥10%, PGA ≥3	Tildrakizumab 200 mg at weeks 0 and 4, then Q12W	49.0 (11.6)	71.4 (13.1)	NR	NR	Japanese: 100
				Tildrakizumab 100 mg at weeks 0 and 4, then Q12W	46.3 (11.9)	68.4 (14.7)	NR	NR	Japanese: 100
				Placebo	50.5 (12.6)	69.2 (14.0)	NR	NR	Japanese: 100
Seo^18^ 2021 (3)	62	12	PASI ≥12, BSA ≥10%, sPGA ≥3	Brodalumab 210 mg at weeks 0 and 1, then Q2W	43.5 (14.3)	71.0 (15.0)	10.9	10.0	Korean: 100
				Placebo	43.7 (15.8)	72.8 (13.6)	13.6	36.4	Korean: 100
SustalMM^19^ (2/3)	171	16	PASI ≥12, BSA ≥10%, sPGA ≥3	Risankizumab 150 mg at weeks 0 and 4, then Q12W	53.3 (11.9)	74.1 (16.2)	NR	29	Japanese: 100
				Placebo	50.9 (11.2)	75.1 (17.7)	NR	24	Japanese: 100
Umezawa^20^ 2021 (2/3)	127	16	PASI ≥12, BSA ≥10%, PGA ≥3	Certolizumab pegol 400 mg Q2W	52.4 (11.6)	71.6 (14.3)	13.2	Anti‐TNF: 5.7	Japanese: 100
				Certolizumab pegol 400 mg at weeks 0, 2, and 4, then 200 mg Q2W	48.4 (13.5)	72.6 (14.3)	12.7	Anti‐TNF: 6.3	Japanese: 100
				Placebo	47.9 (11.4)	75.1 (15.8)	12.7	Anti‐TNF: 3.8	Japanese: 100
VOYAGE 1, VOYAGE 2, pooled^21^ (3)	837	16	PASI ≥12, BSA ≥10%, IGA ≥3	Guselkumab 100 mg at weeks 0 and 4, then Q8W	41.2 (12.2)	76.5 (17.43)	15.3	27.7	Taiwanese/Korean: 100
				Adalimumab 80 mg at weeks 0, then 40 mg Q2W	38.1 (10.14)	80.5 (15.68)	12.1	26.7	Taiwanese/Korean: 100
				Placebo	42.6 (11.75)	74.3 (14.32)	12.6	26.7	Taiwanese/Korean: 100
Yang^22^ 2012 (3)	84	10	PASI ≥12, BSA ≥10%	Infliximab 5 mg/kg at weeks 0, 2, and 6, then Q8W	39.4 (12.3)	68.2 (9.2)	16.0	NR	Chinese: 100
				Placebo	40.1 (11.1)	67.4 (9.9)	16.0	NR	Chinese: 100

Abbreviations: BIW, twice weekly; BSA, body surface area; IGA, Investigator's Global Assessment; mIGA, IGA modified version; NMA, network meta‐analysis; NR, not reported; PASI, Psoriasis Area Severity Index; PGA, Physician Global Assessment; Q2/4/8/12 W, every 2/4/8/12 weeks; QW, once weekly; SD, standard deviation; SE, standard error; sPGA, static PGA; TNF, tumor necrosis factor.

^a^ Assumed based on enrollment criteria (i.e., inclusion/exclusion criteria) regarding prior therapies (e.g., anti‐TNF).

### 3.1 Risk of bias

Among the 20 studies included in the NMA, 13 (65%) were judged to have some concerns about the overall risk of bias, while four (20%) were rated as low risk of bias and three (15%) as high risk of bias (Table 3).

**TABLE 3:** Risk of bias.

Trial	Randomization process	Deviations from intended interventions	Missing outcome data	Measurement of the outcome	Selection of the reported results	Overall bias
Asahina 2010	High	Low	Low	Some concerns	Low	High
Cai 2017	Low	Low	Low	Low	Low	Low
CAIN457A2318	Low	Low	Low	Low	Low	Low
CLEAR^11^, ^12^	Some concerns	Some concerns	Low	Some concerns	Some concerns	Some concerns
ERASURE^12^	Low	Low	Low	Low	Low	Low
I1F‐MC‐RHBH	Some concerns	Some concerns	High	High	Some concerns	High
Igarashi 2012	Some concerns	Low	Low	Some concerns	Low	Some concerns
LOTUS	Some concerns	Low	Low	Some concerns	Low	Some concerns
Nakagawa 2016	Some concerns	Low	Low	Some concerns	Some concerns	Some concerns
Ohtsuki 2017	Low	Low	Low	Some concerns	Some concerns	Some concerns
Ohtsuki 2018	Low	Low	Low	Low	Some concerns	Some concerns
PEARL	Low	Low	Low	Some concerns	Low	Some concerns
POETYK PSO‐1^16^	Low	Low	Low	Some concerns	Low	Some concerns
POETYK PSO‐3	Some concerns	Low	Low	Low	Low	Some concerns
RESURFACE 1^23^	Low	Low	Low	Low	Low	Low
Seo 2021	Low	Low	Low	Some concerns	Low	Some concerns
SustaIMM	Some concerns	Some concerns	Low	Some concerns	Low	Some concerns
Umezawa	Low	Low	Low	Some concerns	Low	Some concerns
VOYAGE 1^21^	Low	Low	Low	Some concerns	Some concerns	Some concerns
VOYAGE 2^24^	Low	Low	Low	Some concerns	Some concerns	Some concerns
Yang	Some concerns	Low	Low	High	Some concerns	High

### 3.2 Feasibility assessment results

The study design of most trials was of good quality (i.e., phase 3, double‐blind, controlled trials) and it was assumed that any minor differences in trial design across the included studies would not impact the relative treatment effects. No trial was excluded from the analysis because of poor study quality based on the risk of bias assessment.

In consultation with clinical experts, the authors considered the following variables to be treatment effect modifiers: geography/ethnicity, disease severity, prior biologic exposure, and time point of assessment.

Because of the observed heterogeneity based on geography, trials not conducted in East Asian countries were excluded (i.e., India, Kuwait, and Pakistan). There is a likelihood that ethnicity could influence treatment responses in plaque psoriasis; therefore, studies that enrolled patients based on Asian ethnicity yet did not enroll patients from any Asian countries were also excluded from the NMA. Results reported at the trials' primary endpoints were prioritized. Several methods for imputing missing data were used across trials. Non‐responder imputation (or modified non‐responder imputation) was the preferred method for data included in the NMA, followed by last observation carried forward and, lastly, no imputation.

### 3.3 NMA results

**FIGURE 2:**
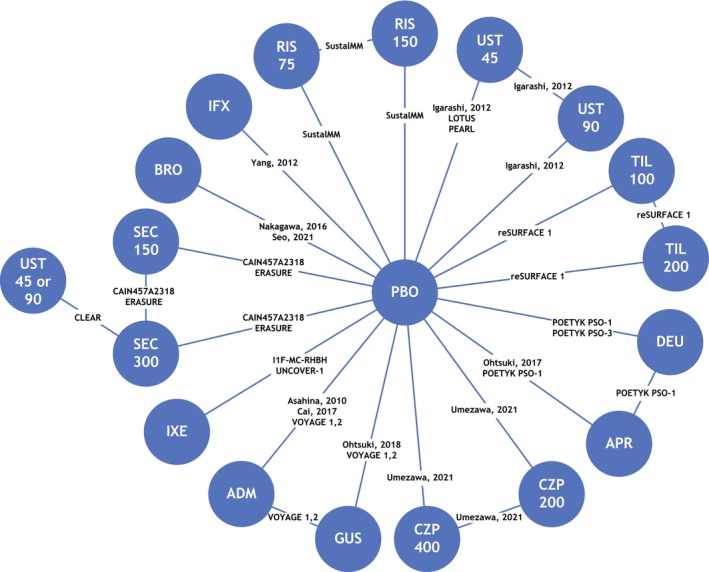
Network diagram. ADM, adalimumab; APR, apremilast; BRO, brodalumab; CZP, certolizumab pegol; DEU, deucravacitinib; GUS, guselkumab; IFX, infliximab; IXE, ixekizumab; PBO, placebo; RIS, risankizumab; SEC, secukinumab; TIL, tildrakizumab; UST, ustekinumab.

The connected network of trials included in the NMA analysis is shown in Figure 2. All treatments included in the analysis were more effective than placebo, and all biologic treatments including deucravacitinib were more effective than apremilast at achieving PASI 75 and PASI 90.

**FIGURE 3:**
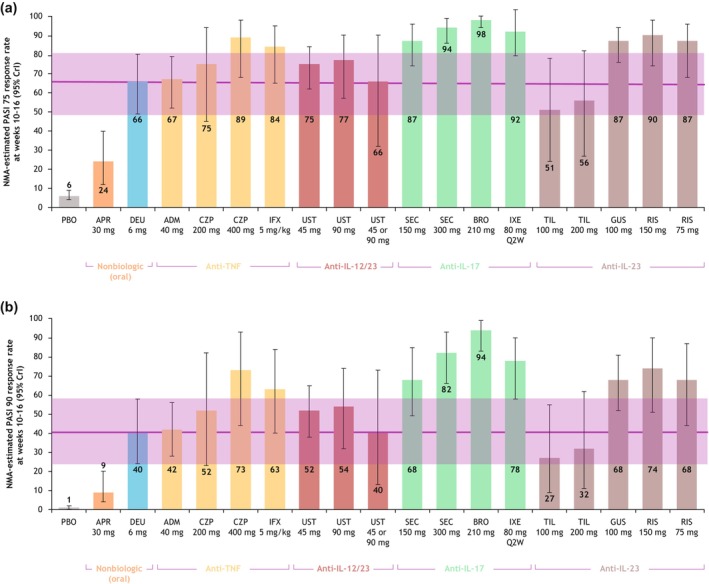
Estimated response rates: (a) Psoriasis Area and Severity Index (PASI) 75; (b) PASI 90. Data are presented as posterior median and 95% credible interval for PASI response rates and were adjusted relative to baseline placebo risk. ADM, adalimumab; APR, apremilast; BRO, brodalumab; CrI, credible interval; CZP, certolizumab pegol; DEU, deucravacitinib; GUS, guselkumab; IFX, infliximab; IL, interleukin; IXE, ixekizumab; NMA, network meta‐analysis; PASI 75, ≥75% reduction from baseline in PASI, 90, ≥90% reduction from baseline in PASI; PBO, placebo; Q2W, every 2 weeks; RIS, risankizumab; SEC, secukinumab; TIL, tildrakizumab; TNF, tumor necrosis factor; UST, ustekinumab.

**FIGURE 4:**
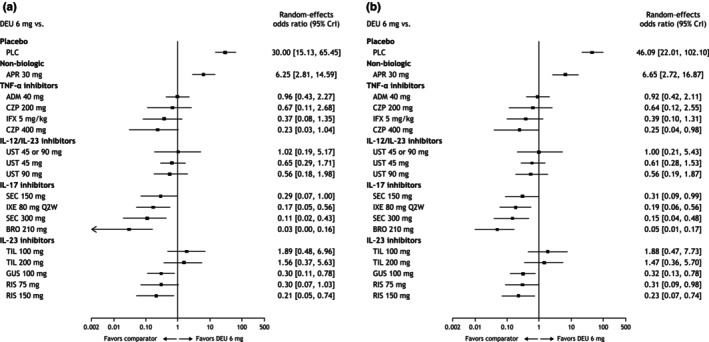
Estimated odds ratios from the network analysis for (a) Psoriasis Area and Severity Index (PASI) 75 and (b) PASI 90. ADM, adalimumab; APR, apremilast; BRO, brodalumab; CrI, credible interval; CZP, certolizumab pegol; DEU, deucravacitinib; GUS, guselkumab; IFX, infliximab; IL, interleukin; IXE, ixekizumab; PASI 75, ≥75% reduction from baseline in PASI, 90, ≥90% reduction from baseline in PASI; PLC, placebo; RIS, risankizumab; SEC, secukinumab; TIL, tildrakizumab; TNF, tumor necrosis factor; UST, ustekinumab.

The NMA‐estimated PASI 75 response rate of deucravacitinib was 66% (95% credible interval [CrI] 49%–80%) in Asian populations, which was notably higher than that for apremilast (24%; 95% CrI 12%–40%; Figure 3a). Based on odds ratios estimated by the NMA, no significant differences were observed between deucravacitinib and the TNF‐α inhibitors adalimumab, certolizumab pegol and infliximab, the IL‐12/23 inhibitor ustekinumab, and the IL‐23 inhibitor tildrakizumab in achieving a PASI 75 response (Figure 4a).

The PASI 90 response rate of deucravacitinib was 40% (95% Crl 24%–58%) in the Asian population, which was notably higher than that for apremilast (9%; 95% CrI 4%–20%) and placebo (1%; 95% CrI 0.8%–2%; Figure 3b). Based on odds ratios estimated in the NMA, no significant differences were observed between deucravacitinib and the TNF‐α inhibitors adalimumab, certolizumab pegol 200 mg, and infliximab, the IL‐12/23 inhibitor ustekinumab, and the IL‐23 inhibitor tildrakizumab in achieving a PASI 90 response (Figure 4b).

Based on odds ratios estimated in the NMA, no significant differences were observed between deucravacitinib and the IL‐17 inhibitors risankizumab (75 mg) or secukinumab (150 mg) in achieving a PASI 50 or PASI 100 response.

**FIGURE 5:**
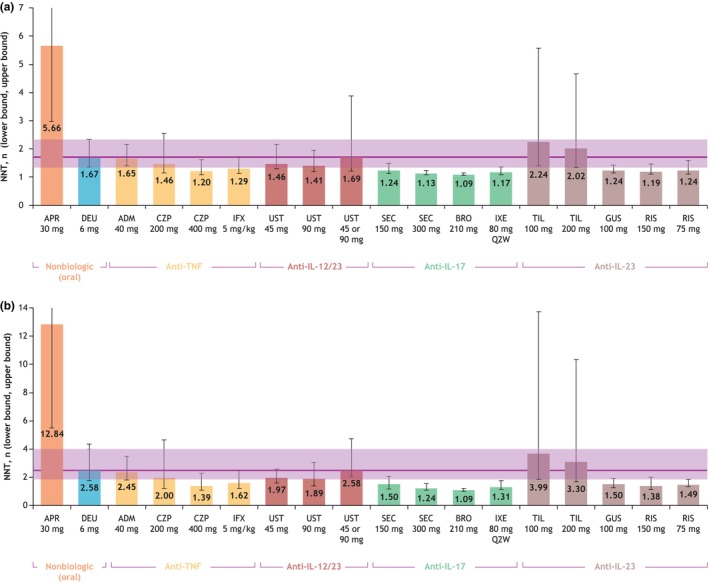
Numbers needed to treat to achieve (a) Psoriasis Area and Severity Index (PASI) 75 response rate and (b) PASI 90 response rate. ADM, adalimumab; APR, apremilast; BRO, brodalumab; CZP, certolizumab pegol; DEU, deucravacitinib; GUS, guselkumab; IFX, infliximab; IL, interleukin; IXE, ixekizumab; PASI 75, ≥75% reduction from baseline in PASI, 90, ≥90% reduction from baseline in PASI; Q2W, every 2 weeks; RIS, risankizumab; SEC, secukinumab; TIL, tildrakizumab; TNF, tumor necrosis factor; UST, ustekinumab.

The NNT to achieve a PASI 75 response rate compared with placebo for deucravacitinib was 1.67, which was substantially less than that for apremilast (5.66). The NNTs for the biologics adalimumab, certolizumab pegol (200 mg), ustekinumab, and tildrakizumab were within the lower and upper bounds for the NNT for deucravacitinib (1.33–2.33) (Figure 5a). The NNT to achieve a PASI 90 response rate compared with placebo for deucravacitinib (2.58) was substantially less than that for apremilast (12.84), and the NNTs for the biologics adalimumab, certolizumab pegol 200 mg, ustekinumab, and tildrakizumab were within the lower and upper bounds of the NNT for deucravacitinib (1.78–4.35) (Figure 5b).

## 4 DISCUSSION

Patients receiving deucravacitinib had a higher probability of achieving PASI 75 and 90 compared with those receiving placebo and apremilast. Deucravacitinib was not significantly different from the anti‐TNFs adalimumab, infliximab, and certolizumab pegol, or the IL‐12/23 inhibitor ustekinumab and the IL‐23 inhibitor tildrakizumab at achieving PASI 75 and 90 responses. These results indicate that the efficacy of deucravacitinib is higher than the non‐biologic apremilast and similar to many available biologic agents. Deucravacitinib was also not significantly different from the IL‐17 inhibitors risankizumab and secukinumab, indicating that the efficacy of deucravacitinib after 10–16 weeks of treatment was similar to that of many available biologic agents. These results are potentially paradigm‐changing because the data demonstrate that a TYK2 inhibitor offers similar efficacy benefits to some biologic therapies.

The findings of this NMA are consistent with those of a recently published global phase 3 NMA, which included trials largely consisting of White patients.^4^ Both analyses found deucravacitinib to be statistically significantly more likely to achieve a higher PASI 75 response than apremilast within 16 weeks of initiating treatment, although the effect was greater in Asian patients (odds ratio = 6.25) than in the global NMA (odds ratio = 2.34). Comparisons with biologics were more favorable for Asian patients than global populations that did not specifically include Asian patients, such that PASI responses for deucravacitinib were comparable to TNF‐α inhibitors adalimumab, infliximab, and certolizumab pegol 200 mg and infliximab, the IL‐12/23 inhibitor ustekinumab, and the IL‐23 inhibitor tildrakizumab.

The results of this NMA should be interpreted in the light of several limitations. Of the 20 trials that reported sufficient information to be included in the NMA, 15 were conducted exclusively in Asian populations enrolled from Asian countries and five trials reported data from an Asian subgroup of a larger, global trial. In some cases, Asian subgroups for which geographies or ethnicities were not specified were presumed, based on the countries from which patients were enrolled. Only five trials had treatment arms with more than 100 patients, and many contained fewer than 50 patients. Comparisons with more than one study were weighted toward those with larger sample sizes. Additionally, data for certain clinically relevant outcomes or treatment differences (effect modifiers) (e.g., disease severity and previous biologic exposure) were limited or not available from all studies. Thus, effect modification testing and subgroup analyses could not be performed. Longer‐term data are important for psoriasis treatments; however, few studies with long‐term endpoints that met the inclusion criteria were identified and, therefore, a robust statistical analysis could not be conducted.

## 5 CONCLUSIONS

Deucravacitinib demonstrated robust efficacy in the East Asian population, with PASI 75 and PASI 90 response rates higher than those for the non‐biologic apremilast and comparable to those of several biologics. The NNT to achieve PASI 75 and PASI 90 with deucravacitinib was substantially lower than that of apremilast and was comparable to that of some biologic therapies. Deucravacitinib provides a convenient oral therapy option with efficacy similar to that of several biologic therapies.

## CONFLICT OF INTEREST STATEMENT

Tsen‐Fang Tsai has served as a clinical trial investigator or consultant with honoraria from AbbVie, AnaptysBio, Boehringer Ingelheim, Bristol Myers Squibb, Celgene, Galderma, GSK, Janssen‐Cilag, Leo Pharma, Lilly, Merck, Novartis, Pfizer, PharmaEssentia, Sanofi, Sun Pharma, and UCB. Yayoi Tada has received research grants from AbbVie, Amgen, Boehringer Ingelheim, Bristol Myers Squibb, Eisai, Jimro, Kyowa Kirin, Leo Pharma, Lilly, Maruho, Sun Pharma, Taiho Pharmaceutical, Tanabe‐Mitsubishi, Torii Pharmaceutical, and UCB; and honoraria from AbbVie, Amgen, Boehringer Ingelheim, Bristol Myers Squibb, Eisai, Janssen, Jimro, Kyowa Kirin, Leo Pharma, Lilly, Maruho, Novartis, Pfizer, Sun Pharma, Taiho Pharmaceutical, Tanabe‐Mitsubishi, Torii Pharmaceutical, and UCB; and consulting fees from AbbVie, Boehringer Ingelheim, Bristol Myers Squibb, Janssen, Lilly, Maruho, Novartis, Taiho Pharmaceutical, and UCB; Dr. Tada was not associated with the editorial process of this manuscript. Camy Kung, Yichen Zhong, Renata M. Kisa are employees and shareholders of Bristol Myers Squibb. Allie Cichewicz, Katarzyna Borkowska, and Tracy Westley are employees of Evidera, which received funding from Bristol Myers Squibb. Yu‐Huei Huang served as a clinical trial investigator or received honoraria as a consultant and speaker for AbbVie, Bristol Myers Squibb, Celgene, Janssen‐Cilag, Novartis, and Pfizer. Xing‐Hua Gao served as a consultant for AbbVie, AstraZeneca, Boehringer Ingelheim, GSK, Janssen Xian, Lilly, and Novartis. Seong‐Jin Jo served as a clinical trial investigator, advisory board member, consultant, or received research grants or speaker's honoraria from AbbVie, Boehringer Ingelheim, Bristol Myers Squibb, Celltrion Healthcare, Daewoong, Green Cross Laboratories, Janssen, Kolon Pharma, Leo Pharma, Lilly, Novartis, Pfizer, Sanofi, UCB, and Yuhan. April W. Armstrong served as a research investigator, scientific advisor, and/or speaker for AbbVie, Almirall, Arcutis, Aslan Pharmaceuticals, Beiersdorf, Boehringer Ingelheim, Bristol Myers Squibb, Dermavant, Dermira, EPI Health, Incyte, Janssen, Leo Pharma, Lilly, Mindera Health, Nimbus, Novartis, Ortho Dermatologics, Pfizer, Regeneron, Sanofi, Sun Pharma, and UCB.

## Supporting information


Table S1.
Table S2.
Table S3.
Table S4.
Table S5.
Table S6.
Table S7.

## Data Availability

The Bristol Myers Squibb policy on data sharing may be found at https://www.bms.com/researchers‐and‐partners/independent‐research/data‐sharing‐request‐process.html.
