# Supplementary material for: Indirect comparison of deucravacitinib and other systemic treatments for moderate to severe plaque psoriasis in Asian populations: A systematic literature review and network meta‐analysis
Source: J Dermatol. 2024 Nov 11;51(12):1559–71. doi: 10.1111/1346-8138.17448 (PMC11624152; doi:10.1111/1346-8138.17448)
Supplement: Supplementary file 1 — Table S1. Table S2. Table S3. Table S4. Table S5. Table S6. Table S7. [file JDE-51-1559-s001.docx]

**SUPPORTING INFORMATION**

**Indirect comparison of deucravacitinib and other systemic treatments for moderate to severe plaque psoriasis in Asian populations: A systematic literature review and network meta-analysis**

Tsen-Fang Tsai, MD^1^; Yayoi Tada, MD, PhD^2^; Camy Kung, PhD^3^; Yichen Zhong, PhD^4^; Allie Cichewicz, MSc^5^; Katarzyna Borkowska, MSc^6^; Tracy Westley, MScPH^5^; Renata M. Kisa, MD, IFAAD^7^; Yu-Huei Huang, MD, PhD^8,9^; Xing-Hua Gao, MD, PhD^9^; Seong-Jin Jo, MD, PhD^10^; April W. Armstrong, MD, MPH^11^

^1^National Taiwan University Hospital, Department of Dermatology, Taipei; ^2^Teikyo University School of Medicine, Department of Dermatology, Tokyo; ^3^Bristol Myers Squibb, Global Medical Affairs, Taipei; ^4^Bristol Myers Squibb, World Wide HEOR, Princeton, NJ; ^5^Evidera, Evidence Synthesis, Modeling and Communication, Waltham, MA; ^6^PPD Poland, Evidence Synthesis, Modeling and Communication, Warsaw; ^7^Bristol Myers Squibb, Princeton, NJ; ^8^Chang Ghang Memorial Hospital and Chang Gung University, Department of Dermatology, Taoyuan City; ^9^First Hospital of China Medical University, Department of Dermatology, Shenyang; ^10^Seoul National University College of Medicine, Department of Dermatology, Seoul; ^11^University of California Los Angeles, Los Angeles, CA

**Table S1.** **Embase (via OvidSP) search strategy.**

| Search number | Search terms |
| --- | --- |
| 1 | exp psoriasis/ or psoria$.ti,ab. |
| 2 | BMS-986165.ti,ab. |
| 3 | exp certolizumab pegol/ or (certolizumab pegol or cimzia or CDP870 or CDP-870).ti,ab. |
| 4 | exp adalimumab/ or (adalimumab$ or humira or d2e7).ti,ab. |
| 5 | exp etanercept/ or (etanercept$ or enbrel or embrel or benepali).ti,ab. |
| 6 | exp brodalumab/ or (brodalumab or siliq or kyntheum or KHK4827 or KHK-4827 or AMG-827 or AMG827).ti,ab. |
| 7 | exp ixekizumab/ or (ixekizumab or taltz or ly2439821 or ly-2439821).ti,ab. |
| 8 | exp secukinumab/ or (secukinumab or cosentyx or ain457 or ain-457).ti,ab. |
| 9 | exp guselkumab/ or (guselkumab or tremfya or cnto-1959 or cnto1959).ti,ab. |
| 10 | exp ustekinumab/ or (ustekinumab or stelara or cnto-1275 or cnto1275).ti,ab. |
| 11 | exp tildrakizumab/ or (tildrakizumab or Ilumya or Ilumetri or mk-3222 or mk3222 or sch-900222 or sch900222).ti,ab. |
| 12 | exp risankizumab/ or (risankizumab or bi 655066 or bi655066 or abbv-066 or abbv066).ti,ab. |
| 13 | exp bimekizumab/ or (bimekizumab or UCB4940 or UCB-4940).ti,ab. |
| 14 | exp infliximab/ or (infliximab or remicade or renflexis or inflectra or ca2).ti,ab. |
| 15 | exp apremilast/ or (apremilast or otezla or cc-10004 or cc10004).ti,ab. |
| 16 | exp interleukin 23p19/ or (interleukin 23p19 or interleukin23p19 or interleukin-23p19 or IL-23p19 or IL-23 p19).ti,ab. |
| 17 | exp methotrexate/ or methotrexate.ti,ab. |
| 18 | exp cyclosporine/ or cyclosporine.ti,ab. |
| 19 | exp fumaric acid dimethyl ester/ or dimethyl fumarate.ti,ab. |
| 20 | exp etretin/ or (acitretin or soriatane or neotigason).ti,ab. |
| 21 | interleukin.mp. |
| 22 | biologic$.ti,ab. |
| 23 | exp mirikizumab/ or (Mirikizumab or LY-3074828 or LY3074828).ti,ab. |
| 24 | exp piclidenoson/ or (Piclidenoson or 3-IB-Meca or CF-101 or CF101 or IB-MECA).ti,ab. |
| 25 | or/2-24 |
| 26 | ((random$ and (assign$ or allocate$)) or rct or trial or crossover or cross over or placebo or controlled study or major clinical study).ti,ab. |
| 27 | exp randomized controlled trial/ or exp randomization/ or exp Single Blind Procedure/ or exp Double Blind Procedure/ or exp Crossover Procedure/ or exp placebo/ |
| 28 | ((doubl$ and blind$) or (singl$ and blind$)).ti,ab. |
| 29 | or/26-28 |
| 30 | 1 and 25 and 29 |
| 31 | (animal/ not human/) or nonhuman/ or exp animal experiment/ or exp experimental animal/ or animal model/ or exp rodent/ or (rat or rats or mouse or mice).ti. |
| 32 | review.pt. not (((systematic or meta) and analy$) or ((indirect or mixed) and treatment comparison)).ti,ab. |
| 33 | 31 or 32 |
| 34 | 30 not 33 |
| 35 | limit 34 to english language |
| 36 | limit 35 to (books or chapter or conference abstract or letter or note or short survey) |
| 37 | 35 not 36 |
| 38 | limit 37 to dc="20200801-20211011" |
| 39 | limit 37 to dc="20211011-20221031" |
| 40 | limit 37 to dc="20221031-20230103" |

**Table S2. MEDLINE (via OvidSP) search strategy.**

| Search number | Search terms |
| --- | --- |
| 1 | exp psoriasis/ or psoria$.ti,ab. |
| 2 | BMS-986165.ti,ab. |
| 3 | exp certolizumab pegol/ or (certolizumab pegol or cimzia or CDP870 or CDP-870).ti,ab. |
| 4 | exp adalimumab/ or (adalimumab$ or humira or d2e7).ti,ab. |
| 5 | exp etanercept/ or (etanercept$ or enbrel or embrel or benepali).ti,ab. |
| 6 | exp brodalumab/ or (brodalumab or siliq or kyntheum or KHK4827 or KHK-4827 or AMG-827 or AMG827).ti,ab. |
| 7 | exp ixekizumab/ or (ixekizumab or taltz or ly2439821 or ly-2439821).ti,ab. |
| 8 | exp secukinumab/ or (secukinumab or cosentyx or ain457 or ain-457).ti,ab. |
| 9 | exp guselkumab/ or (guselkumab or tremfya or cnto-1959 or cnto1959).ti,ab. |
| 10 | exp ustekinumab/ or (ustekinumab or stelara or cnto-1275 or cnto1275).ti,ab. |
| 11 | exp tildrakizumab/ or (tildrakizumab or Ilumya or Ilumetri or mk-3222 or mk3222 or sch-900222 or sch900222).ti,ab. |
| 12 | exp risankizumab/ or (risankizumab or bi 655066 or bi655066 or abbv-066 or abbv066).ti,ab. |
| 13 | exp bimekizumab/ or (bimekizumab or UCB4940 or UCB-4940).ti,ab. |
| 14 | exp infliximab/ or (infliximab or remicade or renflexis or inflectra or ca2).ti,ab. |
| 15 | exp apremilast/ or (apremilast or otezla or cc-10004 or cc10004).ti,ab. |
| 16 | exp interleukin 23p19/ or (interleukin 23p19 or interleukin23p19 or interleukin-23p19 or IL-23p19 or IL-23 p19).ti,ab. |
| 17 | exp methotrexate/ or methotrexate.ti,ab. |
| 18 | exp cyclosporine/ or cyclosporine.ti,ab. |
| 19 | exp fumaric acid dimethyl ester/ or dimethyl fumarate.ti,ab. |
| 20 | exp etretin/ or (acitretin or soriatane or neotigason).ti,ab. |
| 21 | interleukin.mp. |
| 22 | biologic$.ti,ab. |
| 23 | exp mirikizumab/ or (Mirikizumab or LY-3074828 or LY3074828).ti,ab. |
| 24 | exp piclidenoson/ or (Piclidenoson or 3-IB-Meca or CF-101 or CF101 or IB-MECA).ti,ab. |
| 25 | or/2-24 |
| 26 | ((random$ and (assign$ or allocate$)) or rct or trial or crossover or cross over or placebo or controlled study or major clinical study).ti,ab. |
| 27 | exp randomized controlled trial/ or exp randomization/ or exp Single Blind Procedure/ or exp Double Blind Procedure/ or exp Crossover Procedure/ or exp placebo/ |
| 28 | ((doubl$ and blind$) or (singl$ and blind$)).ti,ab. |
| 29 | or/26-28 |
| 30 | 1 and 25 and 29 |
| 31 | (animal/ not human/) or nonhuman/ or exp animal experiment/ or exp experimental animal/ or animal model/ or exp rodent/ or (rat or rats or mouse or mice).ti. |
| 32 | review.pt. not (((systematic or meta) and analy$) or ((indirect or mixed) and treatment comparison)).ti,ab. |
| 33 | 31 or 32 |
| 34 | 30 not 33 |
| 35 | limit 34 to English language |
| 36 | limit 35 to (books or chapter or conference abstract or letter or note or short survey) |
| 37 | 35 not 36 |
| 38 | limit 37 to dt="20200801-20211011" |
| 39 | limit 37 to dt="20211011-20221031" |
| 40 | limit 37 to dt="20221031-20230103" |

**Table S3. CENTRAL (via OvidSP) search strategy.**

| Search number | Search terms |
| --- | --- |
| 1 | exp psoriasis/ or psoria$.ti,ab. |
| 2 | BMS-986165.ti,ab. |
| 3 | exp certolizumab pegol/ or (certolizumab pegol or cimzia or CDP870 or CDP-870).ti,ab. |
| 4 | exp adalimumab/ or (adalimumab$ or humira or d2e7).ti,ab. |
| 5 | exp etanercept/ or (etanercept$ or enbrel or embrel or benepali).ti,ab. |
| 6 | (brodalumab or siliq or kyntheum or KHK4827 or KHK-4827 or AMG-827 or AMG827).ti,ab. |
| 7 | (ixekizumab or taltz or ly2439821 or ly-2439821).ti,ab. |
| 8 | (secukinumab or cosentyx or ain457 or ain-457).ti,ab. |
| 9 | (guselkumab or tremfya or cnto-1959 or cnto1959).ti,ab. |
| 10 | exp ustekinumab/ or (ustekinumab or stelara or cnto-1275 or cnto1275).ti,ab. |
| 11 | (tildrakizumab or Ilumya or Ilumetri or mk-3222 or mk3222 or sch-900222 or sch900222).ti,ab. |
| 12 | (risankizumab or bi 655066 or bi655066 or abbv-066 or abbv066).ti,ab. |
| 13 | (bimekizumab or UCB4940 or UCB-4940).ti,ab. |
| 14 | exp infliximab/ or (infliximab or remicade or renflexis or inflectra or ca2).ti,ab. |
| 15 | (apremilast or otezla or cc-10004 or cc10004).ti,ab. |
| 16 | exp Interleukin-23 Subunit p19/ or (interleukin 23p19 or interleukin23p19 or interleukin-23p19 or IL-23p19 or IL-23 p19).ti,ab. |
| 17 | exp methotrexate/ or methotrexate.ti,ab. |
| 18 | exp cyclosporine/ or cyclosporine.ti,ab. |
| 19 | exp Dimethyl Fumarate/ or dimethyl fumarate.ti,ab. |
| 20 | exp Acitretin/ or (acitretin or soriatane or neotigason).ti,ab. |
| 21 | interleukin.mp. |
| 22 | biologic$.ti,ab. |
| 23 | (Mirikizumab or LY-3074828 or LY3074828).ti,ab. |
| 24 | (Piclidenoson or 3-IB-Meca or CF-101 or CF101 or IB-MECA).ti,ab. |
| 25 | or/2-24 |
| 26 | ((random$ and (assign$ or allocate$)) or rct or trial or crossover or cross over or placebo or controlled study or major clinical study).ti,ab. |
| 27 | exp randomized controlled trial/ or exp random allocation/ or exp Single-Blind Method/ or exp Double-Blind Method/ or exp Crossover Studies/ or exp placebos/ |
| 28 | ((doubl$ and blind$) or (singl$ and blind$)).ti,ab. |
| 29 | or/26-28 |
| 30 | 1 and 25 and 29 |
| 31 | (animals/ not humans/) or nonhuman/ or exp Animal Experimentation/ or exp Disease Models, Animal/ or exp rodent/ or (rat or rats or mouse or mice).ti. |
| 32 | review.pt. not (((systematic or meta) and analy$) or ((indirect or mixed) and treatment comparison)).ti,ab. |
| 33 | 31 or 32 |
| 34 | 30 not 33 |
| 35 | limit 34 to English language |
| 36 | limit 35 to up="202008-202110" |
| 37 | limit 35 to up="202110-202210" |
| 38 | limit 35 to up="202210-202301" |

**Table S4. CDSR (via OvidSP) search strategy.**

| Search number | Search terms |
| --- | --- |
| 1 | psoria$.ti,ab. |
| 2 | BMS-986165.ti,ab. |
| 3 | (certolizumab pegol or cimzia or CDP870 or CDP-870).ti,ab. |
| 4 | (adalimumab$ or humira or d2e7).ti,ab. |
| 5 | (etanercept$ or enbrel or embrel or benepali).ti,ab. |
| 6 | (brodalumab or siliq or kyntheum or KHK4827 or KHK-4827 or AMG-827 or AMG827).ti,ab. |
| 7 | (ixekizumab or taltz or ly2439821 or ly-2439821).ti,ab. |
| 8 | (secukinumab or cosentyx or ain457 or ain-457).ti,ab. |
| 9 | (guselkumab or tremfya or cnto-1959 or cnto1959).ti,ab. |
| 10 | (ustekinumab or stelara or cnto-1275 or cnto1275).ti,ab. |
| 11 | (tildrakizumab or Ilumya or Ilumetri or mk-3222 or mk3222 or sch-900222 or sch900222).ti,ab. |
| 12 | (risankizumab or bi 655066 or bi655066 or abbv-066 or abbv066).ti,ab. |
| 13 | (bimekizumab or UCB4940 or UCB-4940).ti,ab. |
| 14 | (infliximab or remicade or renflexis or inflectra or ca2).ti,ab. |
| 15 | (apremilast or otezla or cc-10004 or cc10004).ti,ab. |
| 16 | (interleukin 23p19 or interleukin23p19 or interleukin-23p19 or IL-23p19 or IL-23 p19).ti,ab. |
| 17 | methotrexate.ti,ab. |
| 18 | cyclosporine.ti,ab. |
| 19 | dimethyl fumarate.ti,ab. |
| 20 | (acitretin or soriatane or neotigason).ti,ab. |
| 21 | interleukin.mp. |
| 22 | biologic$.ti,ab. |
| 23 | (Mirikizumab or LY-3074828 or LY3074828).ti,ab. |
| 24 | (Piclidenoson or 3-IB-Meca or CF-101 or CF101 or IB-MECA).ti,ab. |
| 25 | or/2-25 |
| 26 | 1 and 26 |
| 27 | 202008$.up. or 202009$.up. or 202010$.up. or 202011$.up. or 202012$.up. or 2021$.up. |
| 28 | 26 and 27 |

**Table S5. PsycINFO (via EBSCOhost) search strategy.**

| Search number | Search terms |
| --- | --- |
| 1 | MA psoriasis OR TI psoria* OR AB psoria* |
| 2 | TI BMS-986165 OR AB BMS-986165 |
| 3 | MA certolizumab pegol OR TI ( certolizumab pegol or cimzia or CDP870 or CDP-870 ) OR AB ( certolizumab pegol or cimzia or CDP870 or CDP-870 ) |
| 4 | MA adalimumab OR TI ( adalimumab$ or humira or d2e7 ) OR AB ( adalimumab$ or humira or d2e7 ) |
| 5 | MA etanercept OR TI ( etanercept$ or enbrel or embrel or benepali ) OR AB ( etanercept$ or enbrel or embrel or benepali ) |
| 6 | TI ( brodalumab or siliq or kyntheum or KHK4827 or KHK-4827 or AMG-827 or AMG827 ) OR AB ( brodalumab or siliq or kyntheum or KHK4827 or KHK-4827 or AMG-827 or AMG827 ) |
| 7 | TI ( ixekizumab or taltz or ly2439821 or ly-2439821 ) OR AB ( ixekizumab or taltz or ly2439821 or ly-2439821 ) |
| 8 | TI ( secukinumab or cosentyx or ain457 or ain-457 ) OR AB ( secukinumab or cosentyx or ain457 or ain-457 ) |
| 9 | TI (guselkumab or tremfya or cnto-1959 or cnto1959) OR AB (guselkumab or tremfya or cnto-1959 or cnto1959) |
| 10 | MA ustekinumab OR TI ( ustekinumab or stelara or cnto-1275 or cnto1275 ) OR AB ( ustekinumab or stelara or cnto-1275 or cnto1275 ) |
| 11 | TI ( tildrakizumab or Ilumya or Ilumetri or mk-3222 or mk3222 or sch-900222 or sch900222 ) OR AB ( tildrakizumab or Ilumya or Ilumetri or mk-3222 or mk3222 or sch-900222 or sch900222 ) |
| 12 | TI ( risankizumab or bi 655066 or bi655066 or abbv-066 or abbv066 ) OR AB ( risankizumab or bi 655066 or bi655066 or abbv-066 or abbv066 ) |
| 13 | TI ( bimekizumab or UCB4940 or UCB-4940 ) OR AB ( bimekizumab or UCB4940 or UCB-4940 ) |
| 14 | MA infliximab OR TI ( infliximab or remicade or renflexis or inflectra or ca2 ) OR AB ( infliximab or remicade or renflexis or inflectra or ca2 ) |
| 15 | TI ( apremilast or otezla or cc-10004 or cc10004 ) OR AB ( apremilast or otezla or cc-10004 or cc10004 ) |
| 16 | MA Interleukin-23 Subunit p19 OR TI ( interleukin 23p19 or interleukin23p19 or interleukin-23p19 or IL-23p19 or IL-23 p19 ) OR AB ( interleukin 23p19 or interleukin23p19 or interleukin-23p19 or IL-23p19 or IL-23 p19 ) |
| 17 | MA methotrexate OR TI methotrexate OR AB methotrexate |
| 18 | MA cyclosporine OR TI cyclosporine-a OR AB cyclosporine |
| 19 | MA Dimethyl Fumarate OR TI Dimethyl Fumarate OR AB Dimethyl Fumarate |
| 20 | MA Acitretin OR TI ( acitretin or soriatane or neotigason ) OR AB ( acitretin or soriatane or neotigason ) |
| 21 | TX interleukin |
| 22 | TX biologic* |
| 23 | TI (Mirikizumab or LY-3074828 or LY3074828) OR AB (Mirikizumab or LY-3074828 or LY3074828) |
| 24 | TI (Piclidenoson or 3-IB-Meca or CF-101 or CF101 or IB-MECA) OR AB (Piclidenoson or 3-IB-Meca or CF-101 or CF101 or IB-MECA) |
| 25 | S2 OR S3 OR S4 OR S5 OR S6 OR S7 OR S8 OR S9 OR S10 OR S11 OR S12 OR S13 OR S14 OR S15 OR S16 OR S17 OR S18 OR S19 OR S20 OR S21 OR S22 OR S23 OR S24 |
| 26 | TI ( ((random* and (assign* or allocate*)) or rct or trial or crossover or cross over or placebo or controlled study or major clinical study) ) OR AB ( ((random* and (assign* or allocate*)) or rct or trial or crossover or cross over or placebo or controlled study or major clinical study) ) |
| 27 | MA randomized controlled trial or random allocation or Single-Blind Method or Double-Blind Method or Crossover Studies or placebos |
| 28 | TI ( (double* or single*) AND blind* ) OR AB ( (double* or single*) AND blind* ) |
| 29 | S26 OR S27 OR S28 |
| 30 | S1 AND S25 AND S29 |
| 31 | Limit S30 to English language |
| 32 | 202008$.dp. or 202009$.dp. or 202010$.dp. or 202011$.dp. or 202012$.dp. or 2021$.dp. |
| 33 | 31 and 32 |

**Table S6. Embase (via OvidSP) conference abstract search strategy.**

| Search number | Search terms |
| --- | --- |
| 1 | exp psoriasis/ or psoria$.ti,ab. |
| 2 | BMS-986165.ti,ab. |
| 3 | exp certolizumab pegol/ or (certolizumab pegol or cimzia or CDP870 or CDP-870).ti,ab. |
| 4 | exp adalimumab/ or (adalimumab$ or humira or d2e7).ti,ab. |
| 5 | exp etanercept/ or (etanercept$ or enbrel or embrel or benepali).ti,ab. |
| 6 | exp brodalumab/ or (brodalumab or siliq or kyntheum or KHK4827 or KHK-4827 or AMG-827 or AMG827).ti,ab. |
| 7 | exp ixekizumab/ or (ixekizumab or taltz or ly2439821 or ly-2439821).ti,ab. |
| 8 | exp secukinumab/ or (secukinumab or cosentyx or ain457 or ain-457).ti,ab. |
| 9 | exp guselkumab/ or (guselkumab or tremfya or cnto-1959 or cnto1959).ti,ab. |
| 10 | exp ustekinumab/ or (ustekinumab or stelara or cnto-1275 or cnto1275).ti,ab. |
| 11 | exp tildrakizumab/ or (tildrakizumab or Ilumya or Ilumetri or mk-3222 or mk3222 or sch-900222 or sch900222).ti,ab. |
| 12 | exp risankizumab/ or (risankizumab or bi 655066 or bi655066 or abbv-066 or abbv066).ti,ab. |
| 13 | exp bimekizumab/ or (bimekizumab or UCB4940 or UCB-4940).ti,ab. |
| 14 | exp infliximab/ or (infliximab or remicade or renflexis or inflectra or ca2).ti,ab. |
| 15 | exp apremilast/ or (apremilast or otezla or cc-10004 or cc10004).ti,ab. |
| 16 | exp interleukin 23p19/ or (interleukin 23p19 or interleukin23p19 or interleukin-23p19 or IL-23p19 or IL-23 p19).ti,ab. |
| 17 | exp methotrexate/ or methotrexate.ti,ab. |
| 18 | exp cyclosporine/ or cyclosporine.ti,ab. |
| 19 | exp fumaric acid dimethyl ester/ or dimethyl fumarate.ti,ab. |
| 20 | exp etretin/ or (acitretin or soriatane or neotigason).ti,ab. |
| 21 | interleukin.mp. |
| 22 | biologic$.ti,ab. |
| 23 | exp mirikizumab/ or (mirikizumab or LY-3074828 or LY3074828).ti,ab. |
| 24 | exp piclidenoson/ or (piclidenoson or 3-IB-Meca or CF-101 or CF101 or IB-MECA).ti,ab. |
| 25 | or/2-24 |
| 26 | ((random$ and (assign$ or allocate$)) or rct or trial or crossover or cross over or placebo or controlled study or major clinical study).ti,ab. |
| 27 | exp randomized controlled trial/ or exp randomization/ or exp Single Blind Procedure/ or exp Double Blind Procedure/ or exp Crossover Procedure/ or exp placebo/ |
| 28 | ((doubl$ and blind$) or (singl$ and blind$)).ti,ab. |
| 29 | or/26-28 |
| 30 | 1 and 25 and 29 |
| 31 | (animal/ not human/) or nonhuman/ or exp animal experiment/ or exp experimental animal/ or animal model/ or exp rodent/ or (rat or rats or mouse or mice).ti. |
| 32 | review.pt. not (((systematic or meta) and analy$) or ((indirect or mixed) and treatment comparison)).ti,ab. |
| 33 | 31 or 32 |
| 34 | 30 not 33 |
| 35 | limit 34 to english language |
| 36 | American Academy of Dermatology.cf,cg. |
| 37 | British Association of Dermatologists.cf,cg. |
| 38 | 35 and (36 or 37) |
| 39 | limit 38 to yr="2019 -Current" |
| 40 | limit 39 to dc="20200801-20211011" |
| 41 | limit 38 to dc="20211011-20221031" |
| 42 | limit 38 to dc="20221031-20230103" |

**Table S7. PRISMA Checklist**

| **Section and Topic** | **Item #** | **Checklist item** | **Location where item is reported** |
| --- | --- | --- | --- |
| **TITLE** | | | |
| Title | 1 | Identify the report as a systematic review. Described as network meta-analysis. | Title |
| **ABSTRACT** | | | |
| Abstract | 2 | See the PRISMA 2020 for Abstracts checklist. |  |
| **INTRODUCTION** | | | |
| Rationale | 3 | Describe the rationale for the review in the context of existing knowledge. | Pg 4 |
| Objectives | 4 | Provide an explicit statement of the objective(s) or question(s) the review addresses. | Pg 5 |
| **METHODS** | | | |
| Eligibility criteria | 5 | Specify the inclusion and exclusion criteria for the review and how studies were grouped for the syntheses. | Supporting Table S7 |
| Information sources | 6 | Specify all databases, registers, websites, organizations, reference lists and other sources searched or consulted to identify studies. Specify the date when each source was last searched or consulted. | Pg 7; Supporting Tables S1-S6 |
| Search strategy | 7 | Present the full search strategies for all databases, registers, and websites, including any filters and limits used. | Supporting Tables S1-S6 |
| Selection process | 8 | Specify the methods used to decide whether a study met the inclusion criteria of the review, including how many reviewers screened each record and each report retrieved, whether they worked independently, and if applicable, details of automation tools used in the process. | Pg 5-6 |
| Data collection process | 9 | Specify the methods used to collect data from reports, including how many reviewers collected data from each report, whether they worked independently, any processes for obtaining or confirming data from study investigators, and if applicable, details of automation tools used in the process. | Pg 6 |
| Data items | 10a | List and define all outcomes for which data were sought. Specify whether all results that were compatible with each outcome domain in each study were sought (e.g. for all measures, time points, analyses), and if not, the methods used to decide which results to collect. | Pg 6-7 |
|  | 10b | List and define all other variables for which data were sought (e.g. participant and intervention characteristics, funding sources). Describe any assumptions made about any missing or unclear information. | Pg 5-6 |
| Study risk of bias assessment | 11 | Specify the methods used to assess risk of bias in the included studies, including details of the tool(s) used, how many reviewers assessed each study and whether they worked independently, and if applicable, details of automation tools used in the process. | Pg 8 |
| Effect measures | 12 | Specify for each outcome the effect measure(s) (e.g. risk ratio, mean difference) used in the synthesis or presentation of results. | Pg 6-7 |
| Synthesis methods | 13a | Describe the processes used to decide which studies were eligible for each synthesis (e.g. tabulating the study intervention characteristics and comparing against the planned groups for each synthesis [item #5]). | Pg 6 |
|  | 13b | Describe any methods required to prepare the data for presentation or synthesis, such as handling of missing summary statistics, or data conversions. | Pg 7 |
|  | 13c | Describe any methods used to tabulate or visually display results of individual studies and syntheses. | Pg 8 |
|  | 13d | Describe any methods used to synthesize results and provide a rationale for the choice(s). If meta-analysis was performed, describe the model(s), method(s) to identify the presence and extent of statistical heterogeneity, and software package(s) used. | Pg 7 |
|  | 13e | Describe any methods used to explore possible causes of heterogeneity among study results (e.g. subgroup analysis, meta-regression). | Pg 7 |
|  | 13f | Describe any sensitivity analyses conducted to assess robustness of the synthesized results. | N/A |
| Reporting bias assessment | 14 | Describe any methods used to assess risk of bias due to missing results in a synthesis (arising from reporting biases). | Pg 8 |
| Certainty assessment | 15 | Describe any methods used to assess certainty (or confidence) in the body of evidence for an outcome. | Pg 7 |
| **RESULTS** | | | |
| Study selection | 16a | Describe the results of the search and selection process, from the number of records identified in the search to the number of studies included in the review, ideally using a flow diagram. | Pg 7-8; Supporting Figure S1 |
|  | 16b | Cite studies that might appear to meet the inclusion criteria, but which were excluded, and explain why they were excluded. | Supporting Figure S1 |
| Study characteristics | 17 | Cite each included study and present its characteristics. | Supporting Table S9 |
| Risk of bias in studies | 18 | Present assessments of risk of bias for each included study. | Pg 8; Supporting Table S10 |
| Results of individual studies | 19 | For all outcomes, present, for each study: (a) summary statistics for each group (where appropriate) and (b) an effect estimate and its precision (e.g. confidence/credible interval), ideally using structured tables or plots. | Pg 9-10; Figures 2-3 |
| Results of syntheses | 20a | For each synthesis, briefly summarize the characteristics and risk of bias among contributing studies. | Supporting Table S10 |
|  | 20b | Present results of all statistical syntheses conducted. If meta-analysis was done, present for each the summary estimate and its precision (e.g. confidence/credible interval) and measures of statistical heterogeneity. If comparing groups, describe the direction of the effect. | Figures 2-3 |
|  | 20c | Present results of all investigations of possible causes of heterogeneity among study results. | Pg 9 |
|  | 20d | Present results of all sensitivity analyses conducted to assess the robustness of the synthesized results. | N/A |
| Reporting biases | 21 | Present assessments of risk of bias due to missing results (arising from reporting biases) for each synthesis assessed. | Pg 8 |
| Certainty of evidence | 22 | Present assessments of certainty (or confidence) in the body of evidence for each outcome assessed. | Pg 10 |
| **DISCUSSION** | | | |
| Discussion | 23a | Provide a general interpretation of the results in the context of other evidence. | Pg 10 |
|  | 23b | Discuss any limitations of the evidence included in the review. | Pg 11 |
|  | 23c | Discuss any limitations of the review processes used. | Pg 11 |
|  | 23d | Discuss implications of the results for practice, policy, and future research. | Pg 11 |
| **OTHER INFORMATION** | | | |
| Registration and protocol | 24a | Provide registration information for the review, including register name and registration number, or state that the review was not registered. | Not registered |
|  | 24b | Indicate where the review protocol can be accessed, or state that a protocol was not prepared. | Pg 5 |
|  | 24c | Describe and explain any amendments to information provided at registration or in the protocol. | N/A |
| Support | 25 | Describe sources of financial or nonfinancial support for the review, and the role of the funders or sponsors in the review. | Pg 13 |
| Competing interests | 26 | Declare any competing interests of review authors. | Pg 13 |
| Availability of data, code and other materials | 27 | Report which of the following are publicly available and where they can be found: template data collection forms; data extracted from included studies; data used for all analyses; analytic code; any other materials used in the review. | Pg 14 |
